# Auditory Processing of Non-speech Stimuli by Children in Dual-Language Immersion Programs

**DOI:** 10.3389/fpsyg.2021.687651

**Published:** 2021-10-18

**Authors:** Chloe Jones, Elizabeth Collin, Olga Kepinska, Roeland Hancock, Jocelyn Caballero, Leo Zekelman, Maaike Vandermosten, Fumiko Hoeft

**Affiliations:** ^1^Department of Psychological Sciences, University of Connecticut, Storrs, CT, United States; ^2^Department of Psychology, University of Alabama at Birmingham, Birmingham, AL, United States; ^3^Department of Speech, Language and Hearing Sciences, University of Connecticut, Storrs, CT, United States; ^4^Department of Psychiatry and Behavioral Sciences, School of Medicine, University of California, San Francisco, San Francisco, CA, United States; ^5^Department of Cognition, Emotion, and Methods in Psychology, University of Vienna, Vienna, Austria; ^6^Brain and Language Lab, Cognitive Science Hub, University of Vienna, Vienna, Austria; ^7^Department of Behavioral and Cognitive Biology, Faculty of Life Sciences, University of Vienna, Vienna, Austria; ^8^Department of Psychology, Faculty of Psychology and Educational Sciences, University of Geneva, Geneva, Switzerland; ^9^Brain Imaging Research Center, University of Connecticut, Storrs, CT, United States; ^10^Speech and Hearing Bioscience and Technology, Harvard University, Cambridge, MA, United States; ^11^Department of Neurosciences, KU Leuven, Leuven, Belgium; ^12^Departments of Mathematics, Neuroscience, Psychiatry, and Educational Psychology, University of Connecticut, Storrs, CT, United States; ^13^Haskins Laboratories, New Haven, CT, United States

**Keywords:** language development, frequency modulation, rise time, bilingual, multilingual, phonological awareness, temporal, dynamic

## Abstract

Perception of low-level auditory cues such as frequency modulation (FM) and rise time (RT) is crucial for development of phonemic representations, segmentation of word boundaries, and attunement to prosodic patterns in language. While learning an additional language, children may develop an increased sensitivity to these cues to extract relevant information from multiple types of linguistic input. Performance on these auditory processing tasks such as FM and RT by children learning another language is, however, unknown. Here we examine 92 English-speaking 7–8-year-olds in the U.S. and their performance in FM and RT perceptual tasks at the end of their second year in Cantonese or Spanish dual-language immersion compared to children in general English education programs. Results demonstrate that children in immersion programs have greater sensitivity to FM, but not RT, controlling for various factors. The immersion program students were also observed to have better phonological awareness performance. However, individual differences in FM sensitivity were not associated with phonological awareness, a pattern typically observed in monolinguals. These preliminary findings suggest a possible impact of formal language immersion on low-level auditory processing. Additional research is warranted to understand causal relationships and ultimate impact on language skills in multilinguals.

## Introduction

The multilingual population has increased greatly across the world and in the United States (U.S.) (Ryan, 2013). According to the United States Census Bureau (2015), the number of people who speak a language other than English at home has risen by 158% compared to the country’s population that rose by 38% from 1980 to 2011 (Ryan, 2013), reaching 20.7% of the population. Due to increasing needs, dual-language immersion programs have also grown in the U.S. to more than 2000 programs in an effort to support English Learners (ELs) (Watanabe, 2011; Maxwell and Jose, 2012). These programs originally aimed to support ELs during their early years of education by providing formal instruction in their native language while also exposing ELs to English native and balanced bilingual speakers. Due to the increasing emergence of these programs, we may investigate how formal bilingual immersion may be related to processes crucial to language acquisition, such as auditory processing.

Auditory processing skills are essential for language acquisition and language-related skills such as phonological awareness. Intact auditory processing leads to the development of phonological awareness skills via the formation of phonemic representations (Talcott et al., 2000, 2002; Tallal, 2004; Pasquini et al., 2007; Poelmans et al., 2011). Indeed, associations between auditory processing and phonological awareness have been observed in all ages from infants to adults (Boets et al., 2011; Poelmans et al., 2011; Law et al., 2014). Poor auditory processing skills have been linked to language-based learning disabilities, such as decoding-based reading disorder (also known as dyslexia) (Gaab et al., 2007; Iliadou et al., 2009; Hämäläinen et al., 2013), and specific language impairment (SLI) (Richards and Goswami, 2015).

Languages differ across a multitude of acoustic characteristics and dimensions such as differences in stress patterns, phonemic inventory, and phonotactic constraints. This makes their speakers differentially sensitive to various auditory cues, which are for example frequency modulation (FM) (Talcott et al., 2003; Luo et al., 2007; Cabrera et al., 2015), fundamental frequency (Krishnan et al., 2009), and rise time (RT) (Surányi et al., 2009). Many languages have shown a relationship between auditory processing and language skills such as phonological awareness, including Spanish, Cantonese (Goswami et al., 2010), and English (Corriveau et al., 2010), but cross-linguistic studies have yet to be performed. Since there are demonstrated differences in auditory processing between languages (Talcott et al., 2003; Luo et al., 2007; Krishnan et al., 2009; Surányi et al., 2009; Cabrera et al., 2015; Mattsson et al., 2018), it may be hypothesized that sensitivity to different auditory cues, and the relationship between auditory and phonological awareness may vary depending on exposure to additional languages. Therefore, we aim to examine how significant and consistent exposure to an additional language may be related to auditory processing, which in turn may be associated with phonological awareness. We focus on children that are still in the developmentally sensitive period for language acquisition.

Bilingualism provides speakers with more representations of speech sounds and suprasegmental features compared to monolingual speakers. This has been found for phonemic representations (Paradis, 2001), stress and tone perception (Tong et al., 2015), and fundamental frequency (Skoe et al., 2017). Further, this perceptual bilingual advantage has been shown to translate to language skills such as phonological processing (Wang et al., 2005, 2008; Zhang and McBride-Chang, 2010; Tong et al., 2017; but see Bialystok et al., 2003 where no advantage was reported). For example, studies have found a transfer effect of Chinese tone sensitivity (Mandarin/Cantonese) to English/non-tonal phonological awareness in bilingual children (Wang et al., 2008). This transfer effect has been observed even after controlling for English phonological awareness (Wang et al., 2009), vocabulary, morphological awareness, and orthographic processing (Wang et al., 2005). Tong et al. (2017) examined the mechanism underlying the transfer effect and found two pathways. In one pathway, English stress sensitivity was found to mediate the relationship between Cantonese tone sensitivity and English word reading. In the second pathway, the relationship was mediated by segmental phonological awareness transfer between languages. Whether perception of RT, which is integral to English stress perception, is higher in Chinese/English bilingual children has not been investigated, though it is warranted given this literature. This research provides evidence for how prosodic feature processing impacts phonological awareness across languages. In bilinguals, however, two key auditory processing measures—FM and RT—that are important for language skills such as phonological awareness, have yet to be investigated. FM describes the fluctuations of sound frequency over time. There is evidence that when this information is reduced in the speech stream, it disrupts speech intelligibility (Drullman et al., 1994). FM perception has also been demonstrated to be predictive of phonological awareness (Boets et al., 2011). RT, the duration (in ms) for the amplitude to rise to its peak at the envelope onset, has been found to account for 35% of individual variance in phonological awareness after controlling for the effects of short term memory and IQ (Hämäläinen et al., 2005). RT is also an essential cue for perceiving syllable-stress (Greenberg, 2006; Leong et al., 2011).

The present study, therefore aims to corroborate previous findings and expand upon them, by examining differences in non-speech auditory processing and phonological awareness across three groups of English dominant children learning different languages and writing systems in the first years of formal schooling in the U.S.: (1) children in a dual-language immersion program receiving approximately 80% of their instruction in Cantonese, and 20% in English (Cantonese immersion; CantI); (2) children in a dual-language immersion program receiving approximately 80% instruction in Spanish and 20% English (Spanish immersion; SpanI); and (3) children in a general English education program (GenEd) receiving 100% instruction in English. We predict that performance will vary on the different auditory processing tasks (FM and RT) between children exposed to Cantonese, Spanish, and English predominantly at the end of their 2nd year of formal schooling. If the results align with past studies of monolingual speakers, we would expect to see differences in FM and RT discrimination related to language properties, and related to phonological awareness.

According to the prior literature, we make specific hypotheses. **Hypothesis 1:** Regarding FM, there is research that speakers of tonal languages, such as Cantonese, may be more reliant on cues that track frequency (Krishnan et al., 2009; Cabrera et al., 2015). We therefore hypothesize **greater sensitivity to FM in CantI compared to SpanI and GenEd** [**H1].** Our hypotheses heavily rest on prior findings in monolinguals of various languages, and this premise may not be applicable to bilinguals. Auditory processing (FM and RT sensitivity) may therefore be associated with increase in linguistic diversity more broadly. If so, **an alternative hypothesis [H1a] is that those formally learning multiple languages, regardless of the languages (CantI and SpanI), will show greater auditory processing abilities compared to the GenEd group.**

**Hypothesis 2:** Regarding RT, because literature demonstrates a potential transfer of tone perception to English stress sensitivity in tonal language speakers (Wang et al., 2005, 2008, 2009; Tong et al., 2015, 2017), we hypothesize **greater sensitivity to RT in CantI** compared to GenEd **[H2a]**. Concerning the SpanI group, although both English and Spanish are stress-based languages that rely on lexical stress, Spanish relies heavily on stress for lexical meaning and verbal conjugations, which may result in Spanish speakers relying more heavily on cues of stress (Campbell, 2018). In line with this, research has found Spanish speakers to perform better on stress perception tasks compared to English speakers (Ortega-Llebaria et al., 2013; Romanelli and Menegotto, 2015). Therefore, we also hypothesize **greater sensitivity to RT in SpanI** compared to GenEd **[H2b]**. Third, we make **no predictions about RT measures between SpanI and CantI [H2c]**. **Alternatively, SpanI and CantI may both show similarly increased auditory processing abilities compared to the GenEd group.**

**Hypothesis 3:** Due to the broad literature that reports an association between phonological skills and auditory processing in monolinguals of various languages (Talcott et al., 2003; Surányi et al., 2009; Corriveau et al., 2010; Goswami et al., 2010; Vanvooren et al., 2017), we hypothesize **greater sensitivity to auditory processing is associated with better performance on phonological awareness [H3]**.

## Materials and Methods

Ninety-two participants from CantI programs, SpanI programs and GenEd programs were tested at the beginning of kindergarten (Time 1) and the end of first grade (Time 2). They completed three computerized psychoacoustic tasks at Time 2 that require discrimination between non-speech stimuli a) with different FM, b) with different RT and c) different sound intensity (a control task of Intensity Discrimination [ID]) which has not been shown to be related to language skills (Hämäläinen et al., 2013). The control task was aimed to capture variability in performance that may be due to the general cognitive demands of performing any auditory, computerized task. They also completed a battery of language and cognitive measures such as phonological awareness measures and IQ. Though different measures were collected at different time points, and thus we refer to Time 1 and Time 2, the main analysis of this study is cross-sectional.

### Sample Characteristics

Sample characteristics are described in Tables 1A,B. The sample had more males (61.0%) and a mean age of 7.12 years (*SD* = 0.033) at Time 2. The number of languages each participant was exposed to throughout their lifetime ranged from one to five languages, with the most common being two languages (*n* = 58, 62.4%) followed by three languages (*n* = 15, 16.3%). Overall, the participants were exposed to over 20 different languages. Individual language history varied greatly resulting in a highly heterogenous multilingual sample, however, all participants spoke English. Children and their parents were recruited from public school programs including public dual-language immersion programs in the Bay Area of San Francisco in the U.S. Language exposure data was collected via parental self-report and in-person interviews. The data were summarized using the Bilingual Language Experience Calculator (BiLEC, Unsworth, 2013).

**TABLE 1A:** Mean (SD) sample characteristics and analysis of variance at Time 2.

	**GenEd**	**CantI**	**SpanI**	
				***H***

Age	7.19 (0.33)	6.98 (0.31)	7.1 (0.34)	4.00
Non-verbal IQ^a^	101.42 (13.04)	106.06 (13.49)	106.74 (14.35)	3.19
Number of Languages^b^	2.43 (0.93)	2.73 (0.88)	2.16 (0.51)	6.96^*^
PILE^c^	0.15 (0.19)	0.31 (0.30)	0.23 (0.23)	7.35^*^
Phonemic Inventory^d^	75.13 (23.59)	88.33 (20.90)	69.16 (11.48)	17.14^***^

*^a^Matrix Reasoning subtest from the Kaufman Brief Intelligence Test, 2nd Edition (KBIT), collected at Time 1.*

*^b^Number of Languages refers to the total number of languages to which a child was exposed.*

*^c^PILE = Pre-immersion Language Exposure expressed in cumulative years. See “Cumulative Multilingual Exposure Prior to Immersion Program”.*

*^d^Expressed as total number of unique phonemes. See “Phonemic Inventory”.*

*p < 0.05, ***p < 0.001.

**TABLE 1B:** Frequency (percentage of total) of sample characteristics and tests of expected frequencies at Time 2.

		**GenEd**	**CantI**	**SpanI**	
					**χ^2^**

Sex	Male	28 (70.00%)	7 (41.20%)	21 (60.00%)	4.18
	Female	12 (30.00%)	10 (58.8%)	14 (40.00%)	
Tonal exposure^*a*^	Yes	8 (20.00%)	17 (100.00%)	3 (8.60%)	48.82^***^
	No	32 (80.00%)	0 (0.00%)	32 (91.4%)	

*^a^Tonal Exposure refers to the number of participants who had exposure to a tonal language.*

***p< 0.001.

### Neuropsychological Testing

Participants completed the Elision and Blending Words subtests from the English Comprehensive Test of Phonological Processing 2nd Edition (CTOPP-2) (Wagner et al., 2013) to assess phonological awareness at both Time 1 and Time 2. Elision requires participants to eliminate syllables from presented target words increasing in difficulty until participants are eliminating single phonemes. Blending Words requires participants to combine segmented syllables and eventually phonemes into a cohesive word. Participants also completed the Kaufman Brief Intelligence Test, 2nd Edition (KBIT) Matrix Reasoning subtest to assess non-verbal IQ at Time 1 (Carlozzi, 2011). English vocabulary was assessed at both Time 1 and Time 2 by the Peabody Picture Vocabulary Test—4th Edition (PPVT-4) (Dunn et al., 2007). These were included as part of a larger test battery examining children’s language and cognitive development. See Table 2 for the final number of participants with complete and valid data for each neuropsychological assessment.

**TABLE 2:** Final number of participants with complete and valid data for each task.

***N***	**GenEd**	**CantI**	**SpanI**	**Total**
Controlled FM	31	15	27	73
Controlled RT	27	16	27	70
Intensity discrimination (control task)	33	16	29	78
Non-verbal IQ^*a*^	36	16	34	86
English vocab at time 1^*b*^	36	16	35	87
Elision at time 1^*c*^	35	16	34	85
Blending words at time 1^*c*^	35	16	35	86
English vocab at time 2^*b*^	33	16	35	84
Elision at time 2^*c*^	39	17	35	91
Blending words at time 2^*c*^	39	17	34	90

*^a^Matrix Reasoning subtest from the Kaufman Brief Intelligence Test, 2nd Edition (KBIT).*

*^b^Receptive vocabulary score from the Peabody Picture Vocabulary Test (PPVT-4).*

*^c^Subtest from the Comprehensive Tests of Phonological Processing, 2nd Edition (CTOPP-2).*

### Phonemic Inventory

In an attempt to quantify language exposure in this highly heterogenous sample, phonemic inventories were calculated. Rather than simply count the number of languages, this is an additional descriptive metric of language exposure. This number represents the total unique phonemes to which a child is exposed, so a child exposed to linguistically distant languages would have a larger phonemic inventory than a child exposed to linguistically similar languages. This attempts to better describe the diversity of language exposure rather than assume that all languages are equally additive to phonemic exposure. The inventories were calculated using cross-linguistic data from PHOIBLE 2.0 (Moran and McCloy, 2014),^1^ for each child, we calculated their total phonemic inventory representing the sum of unique phonemes in all languages to which they were exposed. Language exposure information was collected from parental self-report and in-person interviews (as described above). Calculating phonemic inventories may not necessarily be the optimal method to calculate linguistic diversity as the amount of exposure differs for each language at any given time of their life, so we also calculated cumulative and weighted exposure to different languages (see below).

### Cumulative Multilingual Exposure Prior to Immersion Program

Cumulative length of multilingual exposure is defined in this study as the total time (expressed as a ratio) of all languages other than English to which a child was exposed. We calculated this measure following methods described in Unsworth (2013), and used an adapted version of the ‘‘Amount of language exposure in the past’’ section of the BiLEC form.^2^ In short, the following information was used to calculate the measure: (1) how many and which languages a child has been exposed to so far at home and (if applicable) at daycare; (2) what proportion of time each adult or sibling living in the house spoke English or another language for each 1-year period in the child’s life; (3) if the child attended daycare, how much time they spent there per week and what was the approximate time proportion of languages used for instruction for each 1-year period in the child’s life. This cumulative index from Unsworth (2013) has proven useful in other studies of bilingual acquisition (i.e., Sun et al., 2016; Vender et al., 2016; Haman et al., 2017).

Using these data, a ratio for each language was calculated for each year of a child’s life, weighted by the amount of exposure. The cumulative exposure for each language was calculated by summing the ratios for each year. To control for pre-immersion language experience, only cumulative exposure until entrance into school program was calculated. The final value is expressed as total year(s) of multilingual language exposure (see Table 1A). If a child has never been exposed to any other language except for English, their resulting value would be 0.0 years; meanwhile a child who has had Spanish, French, and Mandarin exposure for 0.3, 0.5, and 0.5 years of their life for example, may have a cumulative value of 1.3 years. In this example, their weighted cumulative exposure (1.3 years) to languages other than English is divided by their total length of life at the time of entrance into immersion program (e.g., 5.2 years old), resulting in a value of 0.25. Values could range from 0 to 1.

### General Procedures for Psychoacoustic Tasks

The computerized psychoacoustic tasks of FM, RT, and ID were identical to that described in Vanvooren et al. (2017). All auditory processing tasks were performed on a Panasonic Toughbook 55. Presentation of stimuli was monoaurally at the right ear through a calibrated Audio Technica M50x Headset. Stimuli were presented at 70dB sound pressure level (SPL) with an inter-stimulus interval of 350ms. All tasks were performed in a quiet room. Feedback was given for correct or incorrect responses. To estimate the most accurate threshold for each task, discrimination performance was determined through an adaptive procedure that utilizes a two-up, one-down adaptive staircase. Trials are scored as correct or incorrect. The trial run terminates after eight reversals with the average of the last four reversals was calculated as the threshold.

### Frequency Modulation-Discrimination

Participants detected 2 Hz sinusoidal FM of a 1 kHz carrier tone with modulation depth varying in a 3-alternative forced-choice “odd-one-out” paradigm with the reference stimulus at 1 kHz. Modulation depth varied logarithmically between 100 and 11 Hz in 12 steps. From 11 Hz a step size of 1 Hz was used. The reference stimulus was a pure-tone of 1 kHz. The duration of both the reference and the target stimulus was 1,000 ms including 50 ms cosine-gated onset and offset (Vanvooren et al., 2017). The discrimination threshold is the minimum depth of frequency deviation that is needed to detect change in modulation.

### Rise Time-Discrimination

Participants detected speech weighted noises with linear amplitude RTs. RT of stimuli vary logarithmically between 15 and 699 ms in 50 steps. With ABX forced-choice paradigm, threshold was defined as the minimum difference detected between the reference and target stimulus RT.

### Intensity Discrimination

Similar to RT, participants were asked to identify the stimulus differing from the reference with an ABX forced-choice paradigm, discrimination threshold was defined as the minimum ID in dB SPL. Intensity varied linearly between 70 and 80 dB SPL in 40 steps.

## Results

### Demographics and Behavioral Characteristics

To ensure that results were not driven by differences in group characteristics unrelated to language exposure, we tested whether age (*H*_2_, _87__=_ 4.00, *p* = 0.14), sex (χ^2^_2_, _92__=_ 1.48, *p* = 0.14), or general intelligence assessed by a performance IQ/non-verbal reasoning measure (Kaufman Brief Intelligence Test, 2nd Edition Matrix Reasoning subtest) differed between the groups (*H*_2_, _85__=_ 3.19, *p* = 0.20). We also tested English vocabulary as a measure of English proficiency at both Time 1 (*H*_2_, _85_ = 2.06, *p* = 0.36) and Time 2 (*F*_2_, _81_ = 1.36, *p* = 0.26). We did not find any of these measures to vary significantly across groups (see Tables 1A,B, 3).

**TABLE 3:** Mean (SD) performance on neuropsychological tests.

		**GenEd**	**CantI**	**SpanI**	
**Time 1**					***H***

	English vocab^a^	119.31 (13.26)	114.13 (16.42)	117.43 (19.27)	2.06
	Elision^b^	10.40 (2.05)	10.63 (2.16)	10.65 (1.69)	1.80
					***F***
	Blending words^b^	8.86 (2.16)	9.00 (2.81)	9.74 (1.87)	1.57
	Average scaled CTOPP Scores^c^	9.63 (1.80)	9.81 (2.21)	10.19 (1.55)	0.87
**Time 2**					***F***
	English vocab^a^	116.73 (15.40)	113.44 (13.92)	120.34 (13.72)	0.55
	Elision^b^	9.51 (2.79)	9.24 (2.14)	10.76 (3.13)	2.43
	Blending words^b^	10.56 (2.46)	11.59 (2.45)	12.06 (2.69)	3.27*
	Average scaled CTOPP scores^c^	10.04 (2.24)	10.41 (2.13)	11.39 (2.51)	3.68*

*^a^Standardized Scores from the Peabody Picture Vocabulary Test, Fourth Edition (PPVT–4).*

*^b^Subtest Scaled Scores from the Comprehensive Tests of Phonological Processing, 2nd edition (CTOPP-2).*

^c^Average of Scaled Scores from Blending Words and Elision from the Comprehensive Tests of Phonological Processing. *p < 0.05.

### Testing Hypotheses 1 and 2

To test hypotheses 1 and 2, we first removed outliers in the lower bound of performance (z-score ≥ 1.96, *n* = 12) from FM (*n* = 2), from RT (*n* = 4), and ID (*n* = 7) and/or incomplete data from FM (*n* = 4), RT (*n* = 4), and ID (*n* = 7), leaving 74 of the 92 participants. Outliers were removed to account for abnormally poor performance due to lack of effort or attention. If the threshold on the control ID task, had a z-score greater than 1.96, the subject’s values for RT and FM were also excluded. The mean number of trials completed for ID was 38.56 (*SD* = 10.04), for FM was 33.09 (*SD* = 6.77), and for RT 38.60 (*SD* = 9.65). See Table 2 for the final number of participants with complete and valid data for each task.

We then controlled FM and RT measures for age to account for maturation of auditory processing during development (Shafer et al., 2000; Skoe et al., 2015) by creating unstandardized residuals using linear regression and adding them to the mean. Using this method, the FM and RT measures were also controlled for the control task, ID (Law et al., 2014). Henceforth, FM and RT controlled for both age and ID will be referred to as controlled FM and controlled RT. Linear modeling of FM and RT with fixed factor of school program and covariates of age and ID resulted in non-normally distributed residuals and unequal group sizes, so a non-parametric test, Kruskal-Wallis one-way analysis of variance (K-W H test) was performed instead of a one-way analysis of variance (ANOVA). Using the controlled FM and RT measures described above, a K-W H test was performed in order to compare mean group performance on perceptual discrimination for FM and RT. *Post hoc* analyses adjusted by Bonferroni correction for multiple comparisons were completed to further investigate specific between-group differences.

### Results Associated With Hypothesis 1

**FIGURE 1:**
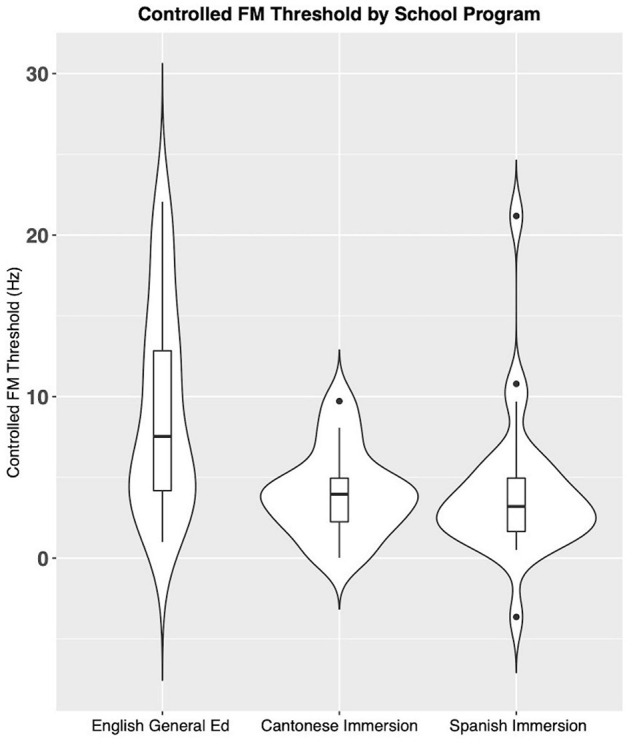
Mean values of controlled FM detection threshold (Hz) across each school program. Lower values indicate better performance. A significant group difference was found (*H*_2_, _72__=_ 15.31, *p* = 0.00047).

The K-W H test revealed a significant effect of school program type for controlled FM performance (*H*_2_, _72__=_ 15.31, *p* = 0.00047 corrected) (Table 4 and Figure 1). Pairwise comparisons indicated that children in CantI had significantly lower detection thresholds (indicating better performance) for controlled FM compared to children in GenEd (*p* = 0.02). This *post hoc* analysis also showed significantly lower controlled FM detection thresholds for children in SpanI compared to children in GenEd (*p* = 0.001). There were no significant differences between SpanI and CantI for controlled FM (*p* = 1.00). These results indicate that children enrolled in dual-language immersion programs have significantly lower detection thresholds for FM compared to children enrolled in GenEd, even after controlling for age and ID performance. These findings confirmed the alternative hypothesis 1 [H1a].

**TABLE 4:** Mean (SD) performance on auditory tasks and analysis of variance.

	**GenEd**	**CantI**	**SpanI**	
				***H***

Controlled FM (Hz)	9.18 (6.30)	3.97 (2.70)	4.07 (4.45)	15.31^***^
Controlled RT (ms)	189.68 (272.50)	258.72 (200.37)	181.73 (311.05)	2.81

***p< 0.001.

### Results Associated With Hypothesis 2

The K-W H test revealed no significant group differences for performance on controlled RT (*H*_2_, _69__=_ 2.81, *p* = 0.25). Mean performances on controlled RT are listed in Table 4. These findings did not confirm our hypotheses of group differences on controlled RT performance.

### Additional Analyses Associated With Hypotheses 1 and 2

In order to elucidate the possible factors driving the significant difference in controlled FM (see section “Results associated with Hypothesis 1”), several aspects of language exposure were investigated to account for the unique characteristics of this multilingual sample. A major component of Hypothesis 1 is that those with exposure to tonal languages would show an increased sensitivity to changes in FM due to the additional perceptual tuning during language exposure.

To account for possible exposure to tonal languages among children in the SpanI and GenEd groups, we considered whether or not any participant had exposure to any tonal language over their lifetime regardless of which type of school they attended. Whether or not languages are tonal was determined with PHOIBLE 2.0 (see text footnote 1; Moran and McCloy, 2014) and *The World Atlas of Language Structures Online* (Maddieson, 2013).^3^ A two-sample permutation *t*-test was performed on FM and RT performance between the groups with and without tonal exposure combining all 3 groups, CantI, SpanI and GenEd groups. Number of permutations were set to 10,000.

To better identify how current language exposure *outside* the immersion programs may be contributing to group differences, a two-sample permutation *t*-test was also performed between those with and without exposure to any tonal language *outside* their immersion program on controlled FM and RT performance, again pooling all participants.

### Additional Results Associated With Hypothesis 1 and 2

Results indicate that lifetime exposure to any tonal language(s) did not drive differences in FM or RT performance across GenEd, CantI, or SpanI groups. There were no significant group differences between children in the tonal language exposed (*n* = 28; 30.40%) and non-exposed (*n* = 64; 69.60%) groups for controlled FM performance (*t* = 1.13, *p* = 0.26), and controlled RT performance (*t* = −0.62, *p* = 0.53) (Table 1B). Additionally, a two-sample permutation *t*-test did not find significant differences between those with (*n* = 18; 19.60%) and without (*n* = 74; 80.4%) concurrent exposure to any tonal language *outside* their immersion program on controlled FM performance (*t* = 0.59, *p* = 0.58), and controlled RT performance (*t* = 0.43, *p* = 0.69). In summary, exposure to tonal languages, whether in general or outside the immersion program, did not result in significant differences on controlled FM performance or controlled RT performance. This finding was unexpected since we expected perceptual tuning related to any tonal language exposure to impact sensitivity at least to FM.

After discovering that tonal language exposure was not a significant differentiating factor for FM or RT performance, but observing that dual-language immersion enrollment regardless of language type *is* a differentiating factor for FM, additional factors related to language exposure were examined. Total phonemic inventory and total number of languages the children were exposed to were investigated. There were no significant correlations found between the size of phonemic inventory and controlled FM (ρ = 0.06, *p* = 0.63) or controlled RT (ρ = 0.06, *p* = 0.63) and no significant correlation found between number of languages exposed prior to entry to school and controlled FM (ρ = 0.13, *p* = 0.27) or controlled RT (ρ = 0.04, *p* = 0.73).

In an attempt to account for pre-immersion language exposure, we calculated cumulative length of multilingual exposure prior to entering the immersion programs (see *Cumulative Multilingual Exposure Prior to Immersion Program*). Controlling for prior language exposure as an unstandardized residual using linear regression did not eliminate our main finding. FM performance across school groups when controlling for age, control task ID, and pre-immersion exposure was still significantly different between the three school groups (*H*_2_, _72_ = 15.11, *p* = 0.001). Pairwise comparisons adjusted with Bonferroni correction for multiple tests still showed the SpanI group’s performance to be significantly better than the GenEd group (*H*_2_, _72_ = 20.50, *p* = 0.001), and the CantI group to be significantly better than the GenEd group (*H*_2_, _72_ = 17.42, *p* = 0.03).

### Testing Hypothesis 3

To further interrogate the significant findings of superior discrimination of FM in the immersion groups (CantI and SpanI) compared to GenEd obtained from the analyses above (see section “Results Associated With Hypothesis 1”), we examined the association between non-speech auditory processing and phonological awareness that has been demonstrated in the literature in monolinguals of various languages. A one-tailed Spearman correlation was performed between controlled FM and a composite score of phonological awareness (an average of CTOPP scaled scores of the subtests Blending Words and Elision). Spearman correlations were implemented given the non-normal distribution of the controlled FM data.

### Results Associated With Hypothesis 3

Based on the results thus far, we examined whether the enhanced auditory processing (greater sensitivity to FM) in the immersion groups (CantI and SpanI) was associated with phonological awareness (average scaled scores of CTOPP subtests). There were no associations between phonological awareness and controlled FM (ρ = −0.10, *p* = 0.41) among the sample combining all three groups. Looking within each group separately, no significant associations between controlled FM and phonological awareness were found: CantI group (ρ = −0.45, *p* = 0.094); GenEd group (ρ = 0.07, *p* = 0.72) or SpanI group (ρ = −0.007, *p* = 0.97). Overall, these findings did not support our hypothesis [H3]. It was found, however, that phonological awareness (average scaled scores of CTOPP subtests) was indeed better in the two immersion groups at Time 2 (CantI and SpanI; Table 3). Furthermore, this significant difference does not appear to be driven by baseline differences between the groups, as no significant differences were found at Time 1 (Table 3).

## Discussion

This paper investigated auditory processing (FM and RT discrimination) of 92 English-dominant first-graders in dual language immersion programs. We aimed to examine whether or not second language acquisition was associated with FM and RT, and whether non-speech auditory processing was associated with phonological awareness skills. Our findings show that children enrolled in dual-language immersion programs were able to better discriminate changes in FM, but not RT, compared to their counterparts in GenEd. Differences in FM were still found even after controlling for age, non-verbal IQ, the control task ID, language exposure prior to entering the immersion program and language exposure outside of the immersion program, indicating that the differences were likely due to formal language exposure in school. After 2 years in each respective language program, a significant difference of English phonological awareness emerged between groups that was not present at the onset of the school programs, with children in dual-language programs showing better phonological awareness. These results could support the idea that formal dual-language exposure in young children facilitates increased sensitivity to fine-grain details of the speech stream in order to process and learn an unfamiliar language However, there still exists the possibility of confounding variables since auditory processing performance was not collected before the onset of program enrollment, but rather, after almost 2 years of enrollment.

This study found CantI students to be better at FM discrimination than the GenEd students, but not significantly better than the SpanI students. These group differences supported hypothesis 1a. They did not support the hypothesis that the CantI would show better FM performance compared to the SpanI group [H1]. The observed increased sensitivity to FM in children enrolled in CantI supports similar results in studies measuring fundamental frequency (F0) in speakers of tonal languages (Krishnan et al., 2009; Skoe et al., 2017). Cabrera et al. (2015) found (tonal) Mandarin speaking adults to be more dependent on FM cues for lexical tone discrimination compared to (non-tonal) French speakers. However, Luo et al. (2007) did not find Chinese speakers to be better at FM discrimination than English speakers. We further demonstrate that greater sensitivity to frequency cues due to Cantonese language exposure is not limited to monolingual adult speakers.

With regards to RT, several studies have pointed to a potential transfer of tonal perception to stress sensitivity (Wang et al., 2005, 2008, 2009; Tong et al., 2015, 2017). We did not, however, find RT discrimination to be significantly enhanced in the CantI program [H2]. Because stress patterns are an important lexical cue for Spanish speakers (Gutiérrez-Palma and Palma Reyes, 2007; Ortega-Llebaria et al., 2013; Campbell, 2018), we anticipated higher RT discrimination performance for the SpanI program, but instead found no significant difference across languages for RT. This may be because all three groups have consistent exposure to English, a lexical stress language, which is also reliant on RT cues, and therefore, all three groups could be similarly sensitive to RT.

Finally, phonological awareness was superior in the immersion groups, but no significant correlations between phonological awareness and FM discrimination were found. Because this sample was chosen for their language experience, and not selected for variability in reading ability, it is possible that the observable differences in FM were not large enough to have an observable benefit in phonological development. This may explain why we did not see a strong replication of the positive relationship of phonological performance to the auditory task performance among the monolingual group that is consistent with the literature (see Hämäläinen et al., 2013 for a review).

What children in immersion programs have in common is exposure to consistent, structured, and formal education in another language, unlike their peers in GenEd. Perhaps this contributes to the differences in auditory processing we observed since children in language immersion programs are more dependent on low-level perceptual cues in order to learn the languages in which they are immersed. Specifically, low-level auditory cues are necessary for the formation of phonemic categories relevant to language production and differentiation of linguistically relevant phonemic contrasts. It is possible that language immersion may extend the period of time to which these children are sensitive to subtle acoustic differences in the speech signal. In fact, previous research has suggested that the critical period of perceptual sensitivity may be extended by bilingual experience (Petitto et al., 2012; Werker and Hensch, 2015). This warrants more investigation as the current study is limited in group heterogeneity related to lifetime language exposure.

### Limitations

Limitations of the study are primarily due to the cross-sectional nature of the auditory task data which preclude thorough analyses of how auditory processing changed before, during, and after immersion. Because there is no auditory task performance data before children entered immersion programs, a causal inference about the effect of immersion cannot be made. Due to this limitation, a timeline describing when differences in auditory processing emerge cannot be assessed. Future studies should evaluate auditory processing pre-immersion and track development over time in larger samples.

Though investigating and controlling for a variety of language experiences, including outside language exposure and prior language exposure, our initial finding of improved FM performance in the immersion groups remained. Despite controlling for these extraneous variables, sample bias cannot be completely ruled out as participants were not randomized to their programs. Although randomizing students to their prospective programs may not be possible in future studies, all efforts to account for sample bias and confounding group differences are encouraged. Without baseline assessments of abilities, it is difficult to ascertain the degree to which self-selection bias may have influenced the results. This potential confound may be particularly pertinent, as families whose child demonstrates early language difficulties may be less likely to opt for a dual-language immersion program.

The high degree of heterogeneity in language history and experience was unexpected in our sample. More precise or accurate data could have been obtained through the use of an environmental language recording device, or with the addition of teacher and staff reports to bolster parental reports. While the highly heterogenous language exposure of our sample provided a highly diverse sample not previously represented in the literature, it makes it challenging to compare these findings to monolingual samples. Additionally, when investigating language acquisition and potential perceptual effects, the age of exposure must be considered. Ultimately, these findings are limited to the non-speech auditory stimuli of FM and RT presented in this study. Current results cannot speak to the broader scope of auditory processing sensitivities that include both speech and non-speech sounds (Vandermosten et al., 2010, 2011; Iliadou et al., 2017; Iliadou and Kiese-Himmel, 2018; Teive et al., 2021). Future research should holistically evaluate auditory processing and conclude if similar effects are observed in older children or adults learning a second language.

## Conclusion

In conclusion, there was a significant group difference in FM performance between the SpanI group, CantI group, and GenEd groups. Students in the SpanI and CantI groups demonstrated greater sensitivity to FM compared to the GenEd group. There was no significant difference found between the SpanI and CantI groups for FM. This finding remained after controlling for previous language exposure and language exposure outside the immersion program. Interestingly, after two years in the school programs, English phonological awareness was greater in the two immersion groups, SpanI and CantI, compared to the GenEd, which was not the case at the onset of schooling, although relationship to FM could not be established.

This study is the first to examine auditory processing in a linguistically diverse sample of children receiving formal dual-language education, thus expanding on previous literature focused only on monolingual populations. Our preliminary findings suggest that low-level auditory processing of non-speech stimuli may be enhanced by formal multilingual immersion.

## Data Availability Statement

The raw data supporting the conclusions of this article will be made available by the authors, without undue reservation.

## Ethics Statement

The studies involving human participants were reviewed and approved by Institutional Review Board at the University of California San Francisco. Written informed consent to participate in this study was provided by the participants’ legal guardian/next of kin.

## Author Contributions

FH, RH, and MV contributed to the conception and design of the study. OK, JC, and LZ collected study data and created study databases. CJ, EC, OK, and FH wrote the manuscript and performed statistical analyses. All authors contributed to manuscript revision.

## Conflict of Interest

The authors declare that the research was conducted in the absence of any commercial or financial relationships that could be construed as a potential conflict of interest.

## Publisher’s Note

All claims expressed in this article are solely those of the authors and do not necessarily represent those of their affiliated organizations, or those of the publisher, the editors and the reviewers. Any product that may be evaluated in this article, or claim that may be made by its manufacturer, is not guaranteed or endorsed by the publisher.

## Footnotes

1
https://phoible.org/

2 The adaptation consisted of extending the form from an assessment of exposure to three different languages to five (as per characteristics of our sample). All other aspects of the “Amount of language exposure in the past” section were used in its original format.

3
https://wals.info/chapter/13
